# Procedure Time and Students' Perception Comparing Full Arch Digital Scans with Conventional Impressions: A Cross-Over Randomized Experimental Trial

**DOI:** 10.1155/2022/6320251

**Published:** 2022-10-17

**Authors:** Hayam A. Alfallaj, Mohammed A. Alsaloum, Sahr H. Altuwaijri, Omar S. Aldibasi, Lubna T. Alkadi

**Affiliations:** ^1^Department of Restorative and Prosthetic Dental Sciences, College of Dentistry, King Saud Bin Abdulaziz University for Health Sciences, Riyadh, Saudi Arabia; ^2^King Abdullah International Medical Research Center (KAIMRC), Riyadh, Saudi Arabia; ^3^Department of Biostatistics and Bioinformatics, King Abdullah International Medical Research Center, Riyadh, Saudi Arabia; ^4^King Saud Bin Abdulaziz University for Health Sciences, Riyadh, Saudi Arabia

## Abstract

**Methods:**

Ninety-six dental students each prepared tooth #36 for an all-ceramic crown on typodont models and were then randomly assigned into either group A: performed digital scan first, or Group B: performed conventional impression first. Procedure time was recorded for both. Immediately following each procedure, students indicated their perceived procedure difficulty. After exposure to both techniques, they selected their preferred one.

**Results:**

There was a statistically significant difference between the mean procedure time of both techniques (*P* < 0.0001), where students spent 663.76 ± 442.50 seconds to complete the conventional impression and 293.32 ± 181.49 seconds to complete the digital scan. Females were significantly faster in completing the conventional impression compared to males. On the contrary, male students were faster in digital scanning than female students. There were no carryover effects in the duration and the initially performed procedure. 76% (73 of 96) of participants preferred digital scanning with no statistical significance shown between the preferred and initially performed procedure. Participants perceived conventional impressions to be more difficult than digital scans. There was a weak positive correlation between the VAS score and the procedure time for the digital technique (*R* = 0.25) and a moderate positive correlation for the conventional technique (*R* = 0.45).

**Conclusions:**

The digital technique was preferred and perceived as easier than the conventional among undergraduate dental students with no impression-making experience, suggesting their readiness for new technology uptake. However, no significant correlation was found between the initially performed procedure and preference.

## 1. Background

Before introducing computer-aided design/computer-aided manufacturing (CAD/CAM) in dentistry, the conventional impression technique was the only available modality to record and reliably duplicate oral tissues. The introduction of the earliest system in 1980, the CEREC system (CEREC; Dentsply Sirona, York, PA), offered many advantages that are relevant in different fields of dentistry, including orthodontics, restorative, and implant dentistry. [1, 2] Intraoral digital scanners capture three-dimensional images of the oral structures which offer numerous benefits over conventional impressions. These include eliminating the risk of impression material distortion, lowering the cost and risk of infection spread, decreasing the working time, in addition to improving patient acceptance. [2] Previous research found that compared to conventional impressions, intraoral scans yielded tooth-supported restorations with comparable internal and marginal fit and led to a consistently better patient acceptance. [3–6] Despite that, some clinicians resist adopting intraoral scanners in their practices, possibly due to their contentment with the first learned impression technique or the demand to invest more time mastering the effective use of this new technology. [3, 4] Clinicians' age and level of expertise are some of the known factors that may influence their preferred impression technique. [3, 4]

The available evidence in implant dentistry suggests that experienced clinicians did not prefer digital scans over conventional vinyl polysiloxane (VPS) impressions to record implant position. [7, 8] In contrast, dental students preferred digital scans and were more efficient when using intraoral scanners to capture the position of a scan body than experienced clinicians. [9, 10] Notably, both groups similarly rated the level of difficulty of using intraoral scanners. [7] However, in the context of orthodontics, although students found alginate impressions to be easier and faster than digital scans, 58% preferred digital impressions over conventional alginate impressions. [11]

In the current years, more dental students are being introduced to intraoral scanners during their undergraduate dental education. [12] In undergraduate dental education, the conventional impression technique is usually taught and practiced before students' exposure to the digital scanning technique. Hence, it would be interesting to know if exposing students first to digital scanning of a single prepared tooth followed by the conventional approach would affect the time required to learn each technique and their perception of their difficulty and preference. To the authors' knowledge, no studies have been conducted to investigate the parameters mentioned above.

The primary aim of this cross-over randomized experimental trial is to test whether the choice of impression technique that is initially practiced on the first preclinical exposure to digital scans or conventional VPS impressions would affect the procedure duration, students' preference, and perceived procedure difficulty level. The secondary aim is to compare procedure duration, students' preference, and perceived procedure difficulty between the two techniques.

## 2. Methods

A cross-over randomized experimental trial was conducted in the preclinical simulation laboratories at the College of Dentistry (COD), King Saud Bin Abdulaziz University for Health Sciences (KSAU-HS), Riyadh, Saudi Arabia. Ethical approval was obtained from King Abdullah International Medical Research Center (KAIMRC) Institutional Review Board (RC20/379/R). The initial sample size was calculated based on the comparison of two independent groups to detect a mean difference of 100 seconds between the two procedures with a standard deviation of 150, alpha = 5%, beta = 10%, and a ratio of 1 : 1. The minimum sample size required was 96 students. Thus, all 104 registered students were enrolled in the study.

### 2.1. Study Setting

One hundred four dental students registered in the preclinical fixed prosthodontics course were included. All the procedures involved in this study were normally performed as part of the approved preclinical fixed prosthodontics course. The participants in this study had no previous experience with any of the impression techniques. Inclusion and exclusion criteria are presented in Table 1.

**Figure 1:**
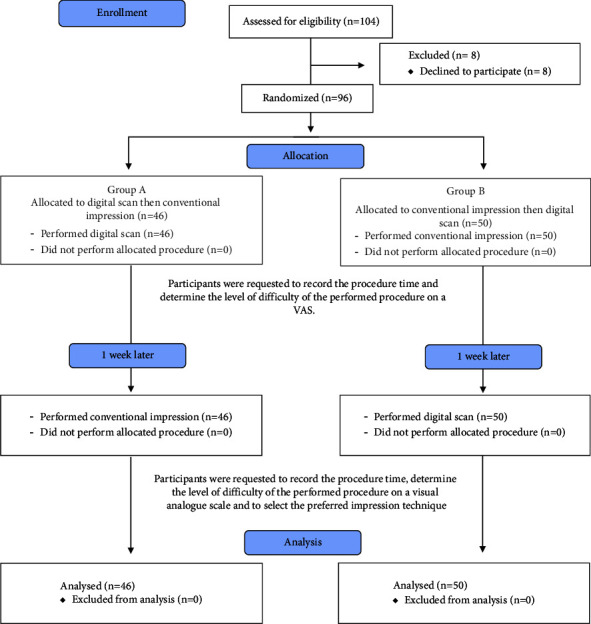
CONSORT 2010 flow diagram.

The study was designed following the Consolidated Standards of Reporting Trials (CONSORT) statement guidelines (Figure 1). Each participant was asked to prepare tooth #36 for a full-coverage lithium disilicate crown on a typodont model with gingival replica (Nissin Dental Product Inc, Kyoto, Japan). Preparations were done according to the required guidelines (1.5 mm reduction occlusally and 1 mm reduction axially with an equigingival deep chamfer finish line). Before impression making, participants received instructions using a PowerPoint presentation. Subsequently, a practical demonstration was given by an experienced prosthodontist, where the step-by-step treatment sequence for both impression techniques was explained. After that, students were randomly assigned (based on their serial numbers) into one of the two groups, Group A: performed a full arch digital scan first, or Group B: performed a full arch conventional impression using VPS first. Students were supervised by two experienced prosthodontists who were calibrated on the required teaching and assessment methods and recorded the procedure time for students in both groups. For both groups, the other impression technique was performed two days thereafter. Immediately following the completion of each impression technique, students were requested to voluntarily fill out a form inquiring about their perceived procedure difficulty. After exposure to both techniques, they were asked to select their preferred one.

### 2.2. Digital Scans

CEREC Omnicam intraoral scanners (CEREC Omnicam; Dentsply Sirona, York, PA) were used. The participants performed the intraoral scanning following the manufacturer's recommendations under the supervision of two experienced prosthodontists who tracked and recorded the procedure time (in seconds) from its initiation until an image of acceptable quality was achieved. The scanned image was deemed acceptable when all the mandibular teeth were wholly and accurately recorded without voids with 2 to 3 mm of soft tissue apical to the gingival margin. The intraoral scanning quality was rejected if the scanned image showed areas of overlap or large voids that the software did not offset. Extra time needed for correction or remaking the impression was added to the recorded procedure time.

### 2.3. Conventional Impressions

Participants were instructed to apply VPS tray adhesive (VPS tray adhesive; 3M ESPE, St. Paul, Minnesota) on custom trays fabricated using Triad®VLC acrylic material (Triad®VLC; Dentsply International, York, PA). A single step impression technique was used, where light body VPS elastomeric impression material (Imprint 3 VPS; 3M ESPE, St. Paul, Minnesota) was injected around the prepared tooth, and the tray was loaded with a heavy body VPS elastomeric impression material (Imprint 3 VPS; 3M ESPE, St. Paul, Minnesota). Materials were handled according to the manufacturer's recommendations. The impression was accepted if all the mandibular teeth were wholly and accurately recorded with no voids or tears around tooth #36 and with 2 to 3 mm of soft tissue apical to the gingival margin. An experienced prosthodontist assessed the produced impressions and recorded the procedure time (in seconds), starting from injecting the light body VPS around the prepared tooth until the impression is removed after it is fully set. When the produced impression was not acceptable, extra time needed for remaking the impression was added to the procedure time.

### 2.4. Preference and Perceived Level of Difficulty

Immediately after their exposure to each of the two impression techniques, students were requested to voluntarily evaluate its difficulty using a Visual Analog Scale (VAS). [13] Students were instructed to put an “X” mark on an uninterrupted 100 mm line to indicate the level of difficulty of the performed impression technique, where 0 indicates not difficult at all and 100 indicates very difficult. After exposure to both impression techniques, participants were asked to select their preferred technique and indicate which technique they would like to start with when they become eligible to treat patients.

### 2.5. Statistical Analysis

Descriptive statistics were used to report participants' gender, initial procedure, procedure time for conventional impressions and digital scans (in seconds), number of repetitions, student preference, and difficulty level using VAS. Comparison between the conventional and digital techniques concerning students' preferences was carried out using chi-square tests. To compare the two procedures, linear mixed-effect models were used for the procedure time and VAS score to test for the carryover effect where the main effects were procedure and gender. Procedure and gender interaction term was included in the models as well. Models' assumptions were verified and met, including homoscedasticity and normality of the error term's distribution. Correlation analysis for the VAS score and the time for each procedure were applied based on the Pearson correlation coefficients. All analyses were conducted using the Statistical Analysis System (SAS) software, version 9.4, (SAS Institute, Inc., Cary, NC, USA). The level of significance was declared at *P* < 0.05.

## 3. Results

Out of 104 second-year dental students registered in the preclinical fixed prosthodontics course, 96 dental students (46 females and 50 males) completed the study form with a response rate of 92.3%, and 52% of participants were males. There was a statistically significant difference between the mean procedure time of both impression techniques (*P* < 0.0001), where students spent 663.76 ± 442.50 seconds on average to complete the conventional impression and 293.32 ± 181.49 seconds to complete the digital scans. When comparing the average procedure time for conventional impressions between male and female students, the female group was significantly faster with an average of 491.33 ± 226.34 seconds spent compared to 822.40 ± 528.18 seconds for their male colleagues (*P* < 0.0001) (Table 2). On the contrary, male students spent significantly less time completing digital impressions than female students, averaging 270.04 ± 170.71 seconds compared to 318.63 ± 191.18 seconds, respectively. According to statistical analysis, there were no carryover effects in the duration and the initially performed procedure. 63% (61 of 96) of students managed to make an acceptable conventional impression on their first attempt. The remaining students repeated the conventional impression at least once, and up to 11 times to meet the impression acceptance criteria. 82% (29 of 35) of students who repeated the conventional impression were males. On the other hand, only 25% (24 of 96) of students repeated the digital scan to meet its acceptance criteria, with two participants repeating the scan twice.

Regarding students' reported preference, 24% (23 of 96) of participants preferred conventional impressions, while 76% (73 of 96) preferred digital scanning. However, there was no statistical significance shown between the preferred and initially performed procedure; 32% (16 of 50) of those who started with the conventional impression preferred the conventional technique. On the other hand, 84.8% (39 of 46) of those who started with the digital scanning preferred the digital technique. Interestingly, 80% (40 of 50) of males preferred digital scanning over conventional impressions. Nevertheless, 33.3% (32 of 96) of students preferred to start with the conventional impression even though 37.5% (12 out 32) of them selected the digital scanning as their preferred technique. Additionally, participants who were more efficient in one impression technique showed preference for the same technique (Table 3).

Participants perceived conventional impressions to be more difficult than digital scans. The mean level of difficultly on VAS scores was 24.94 ± 24.72 for digital impressions and 31.27 ± 26.07 for conventional impressions (*P*=0.0013). Male students reported a significantly higher VAS score compared to female students (*P*=0.0048). Regarding the correlation between the VAS score and the procedure time, there was a weak positive correlation for the digital method (*R* = 0.25) and a moderate positive correlation for the conventional method (*R* = 0.45).

## 4. Discussion

With the numerous advantages offered by the digital workflow and its increased utilization and acceptance, many dental schools are introducing digital scanning technologies in their undergraduate curricula. [12] This study aims to investigate whether starting with digital scanning rather than conventional impressions during students' first encounter with the impression procedure would affect their preference for one technique over the other. Additionally, it compares the procedure duration and perceived procedure difficulty level.

The current study results show that digital scans are more efficient, with a mean procedure time of 293.32 seconds, compared to 663.76 seconds for conventional impressions. Similarly, Bilir and Ayguzen compared the mean procedure time between digital scanning using CEREC Omincam (CEREC Omnicam; Dentsply Sirona, York, PA) and conventional impressions using VPS impression material. They found the mean procedure time to be 272.4 seconds for digital scanning and 639 seconds for conventional impressions. [14] Similar findings were reported in a study by Lee and Gallucci, where they compared conventional impressions and digital scanning to capture implant positions. [10] These findings can be explained by the ease and flexibility of the digital scanning procedure facilitating retake of the missing or overlapped sections without repeating the entire scan. On the contrary, this advantage does not apply to VPS impressions, resulting in a potentially longer duration to obtain an acceptable impression. [15] This also explains why impression remakes were higher in the conventional (37%) than digital (25%) technique.

Although all participants received a demonstration before starting the trial, 37% needed to repeat the conventional impressions at least once. Some were even hesitant to remove the impression material from the typodont model after the setting time recommended by the manufacturer had been exceeded. This resulted in an average of 11 minutes with a broad standard deviation to produce an acceptable conventional impression. Other studies reporting on the average durations for making the same impression may be incomparable to the current study due to variations in participants' experience, working time measurement protocols, and laboratory and clinical settings. [3, 10, 16]

Female students spent less time making conventional impressions compared to male students. In contrast, male students were significantly faster at making digital scans. This significant difference, and the fact that 82% of the students who repeated the conventional impression were males, could be the reason why 40 (80%) male students preferred digital scanning over conventional impressions. In dentistry, students acquire a set of skills in the cognitive, affective, and psychomotor domains. [17] The impact of gender attributes is manifested in students' performance in each of these domains. [18] This can explain the atypical trend in performance between male and female dental students. In a study conducted at a dental program, male students significantly outperformed their female colleagues in cognitive tests, while females significantly outperformed male students in a psychomotor test. [19] Cultural and social factors can further accentuate the influence of gender attributes on students' performance. [20]

Students found digital scanning easier than conventional impressions, with a mean difficulty level of 24.94 on VAS compared to 31.27, respectively. These results are comparable to the study of Joda et al. and Bilir. [7, 14]

The majority (76%) of the students preferred digital scanning, while 24% preferred conventional impressions. Likewise, Lee et al. reported that 60% of students preferred digital scanning, 7% preferred conventional impressions, and 33% had no preference. [8] This may imply that digital scans are more efficient and easier to master, especially for students who have no previous experience with either technique. Therefore, incorporating digital dentistry into the undergraduate dental curriculum may be manageable by students. In contrast, Cheah et al. reported that students with previous clinical experience in conventional impressions who performed digital scanning for the first time in a clinical setting did not demonstrate the same high preference rate for digital scanning. Additionally, they felt significantly more familiar with the conventional impression technique, struggled with maneuvering the bulky scanner intraorally, and experienced difficulties handling the software, leading to an extended working time. [21]

66.7% of the participants indicated that they prefer to start treating patients using digital scanning, which is in line with Bilir and Marti et al.'s studies, where 85% and 96% of all participants indicated their desire to use digital scanning in the future, respectively. [15, 22]

The findings of this study suggest that participants who were more efficient in digital scanning preferred digital impressions and wanted to start treating patients with them. Additionally, participants who preferred to start with conventional impressions were more efficient in making these impressions than those who did not demonstrate the same preference. As digital scanning, like any other procedure, requires learners to go through a learning curve, additional experience and exposure to this technique may result in more students considering it their preferred approach.

In addition to the flexibility and simplicity of digital scanning, operators' experience level is an important factor that influenced the operating time, efficiency, and students' preference. [23, 24] In the current study, the operators were students in their second-year who have no prior experience in any of the techniques. This is similar to the study by Yilmaz et al. where a group of their participants were students in their 4th year with no experience in any of the impression techniques. [23] Experience and familiarity with the technique plays a role even when participants are practicing dentists. In the study by Resende et al., they reported that inexperienced clinicians took longer time, produced lower quality scans, and required more rescanning compared to more experienced clinicians. [24]

Students and younger clinicians are more familiar with new technologies like online applications, computers, and digital software. This might be a possible reason that favored the digital scanning over conventional impression and resulted in less operating time despite the fact the operators are inexperienced in both techniques. [23, 24] Future studies investigating operators' background and the influencing factors would be very helpful to enhance the learning process when integrating new methods and techniques into the undergraduate dental curriculum. However, the current study found no significant carryover between the initially performed procedure and student preference, suggesting that mastering the two techniques is entirely independent and probably requires a different set of skills.

The study utilizes in vitro setting to compare the efficiency and preference of undergraduate students between digital scanning and conventional impressions using a single intraoral scanning system, which are limitations of the current study. The complexity of the clinical setting, with anatomical, behavioral, and patient-related variables may affect the applicability of the results of this study. Moreover, other digital scanning systems may provide variable user experiences based on their features. Future studies comparing conventional impressions to digital scanning in a clinical setting using multiple digital systems are recommended. These will be beneficial to assess whether variable systems would affect the observed results and whether introducing additional layers of complexity such as patient attitude, preference, and the presence of a dental assistant would affect the overall efficiency of the impression system (i.e., reducing operating time and increasing impression accuracy), which in return can influence clinician preferences.

## 5. Conclusions

The digital scanning technique is preferred and perceived as easier than the conventional technique among undergraduate dental students with no impression-making experience, suggesting their readiness for the new technology uptake. However, there was no significant correlation found between the initially performed procedure and student preference. Therefore, integrating digital dentistry into undergraduate dental curricula should be strongly considered to correspond to the considerable increase in digital technology.

## Figures and Tables

**Table 1:** Participants' inclusion and exclusion criteria.

Inclusion criteria	Exclusion criteria
(i) All male and female dental students enrolled in the 2020–2021 preclinical fixed prosthodontics course (ii) Students with no previous experience with both digital and conventional impression techniques	(i) Students who dropped from the course (ii) Unwilling to participate in this study

**Table 2:** Comparison between conventional and digital impressions.

	Conventional impression		Digital impression		*P*-value
Duration (in seconds)	All participants: Mean ± SD 663.76 ± 442.50		All participants: Mean ± SD 293.32 ± 181.49		<0.0001^*∗*^
	Female (46) 491.33 ± 226.34	Male (50) 822.40 ± 528.18	Female (46) 318.63 ± 191.18	Male (50) 270.04 ± 170.71	<0.0001^*∗∗*^

VAS score	All participants: Mean 31.27 ± 26.07		All participants: Mean 24.94 ± 24.72		0.0013^*∗*^
	Female 22.30 ± 19.98	Male 39.52 ± 28.39	Female 26.46 ± 25.16	Male 23.54 ± 24.48	0.0048^*∗∗*^

Preference	All participants: 23 of 96 (24%)		All participants: 73 of 96 (76%)		
	Female 13 of 23 (57%)	Male 10 of 23 (43%)	Female 33 of 73 (45%)	Male 40 of 73 (55%)	0.1803^

The preferred initial procedure	All participants: 32 of 96 (33.3%)		All participants: 64 of 96 (66.7%)		
	Female 15 of 32 (47%)	Male 17 of 32 (53%)	Female 31 of 64 (48%)	Male 33 of 64 (52%)	0.8381^

^*∗*^ *P*-value from the main effect of the mixed model. ^*∗∗*^*P*-value from the interaction term of the mixed model. ^*P*-value from chi-square test.

**Table 3:** Relationship between preferred technique and procedure duration.

Preferred initial clinical procedure	*N*	Duration (in second) Mean ± SD	
		Conventional	Digital
Conventional	32	618.5 ± 353.2	328.1 ± 199.6
Digital	64	686.4 ± 481.9	275.9 ± 170.6

## Data Availability

The datasets used and/or analysed during the current study are available from the corresponding author on reasonable request.

## Abbreviations

CAD/CAM: Computer-aided design/computer-aided manufacturing
CEREC: Chairside economical restorations of esthetic ceramic
COD: College of dentistry
KSAU-HS: King saud bin abdulaziz university for health sciences
SAS: Statistical analysis system
VAS: Visual analog scales
VPS: Vinyl polysiloxane.
